# Efficacy and Safety of Long‐Term Azathioprine Therapy for Severe Alopecia Areata: A 10‐Year Cohort Study

**DOI:** 10.1111/jocd.70187

**Published:** 2025-04-30

**Authors:** Susan Farshi, Parvin Mansouri

**Affiliations:** ^1^ Skin and Stem Cell Research Center Tehran University of Medical Sciences Tehran Iran

**Keywords:** alopecia areata, azathioprine, thiopurine methyltransferase

## Abstract

**Background:**

Alopecia areata is an autoimmune disease in which T cells may play a key role in its pathogenesis. Various immunosuppressive drugs have been employed with varying degrees of success.

**Objectives:**

This study aimed to evaluate the long‐term efficacy and safety of azathioprine as a systemic monotherapy for moderate to severe alopecia areata.

**Methods:**

A total of 63 patients (27 females [42.9%] and 36 males [57.1%]) with a minimum 6‐month history of alopecia areata were included. The extent of scalp hair regrowth was assessed during the treatment and annually for up to 10 years using the Severity of Alopecia Tool (SALT score). The primary endpoint was the percent change in SALT score during treatment. The daily dosage of azathioprine was calculated at 2 mg/kg of body weight.

**Results:**

The mean duration of the current episode of scalp hair loss was 34.10 months (±39.16). The mean percentage of hair regrowth was 92.69% (±9.08). The mean percentage of hair loss decreased from 74.2% (±27.8) before treatment to 5.2% (±8.6) after 10 years of azathioprine treatment, indicating a highly significant statistical improvement (Paired *t*‐test, 95% CI = 55.9–75.3). The mean hair loss score (S0–S5) improved from 5.56 (±1.3) before treatment to 0.67 (±0.53) after 10 years of treatment, showing a significant difference from the baseline score (Wilcoxon signed‐rank test, *p* < 0.0001).

**Conclusion:**

This study demonstrates that azathioprine is a safe and effective systemic therapy for the treatment of recalcitrant and severe alopecia areata.

## 1 Introduction

Alopecia areata (AA) is an immunologically mediated disease characterized by extreme variability in its onset, duration, extent, and pattern of hair loss [1, 2]. It can affect the entire scalp or result in the loss of all body hair [3]. The lifetime risk of developing AA is estimated to be between 1.7% and 2.1% [4, 5].

AA is hypothesized to be an organ‐specific autoimmune disease [6], with genetic predisposition and environmental triggers playing key roles in its development [7, 8, 9]. It is frequently associated with other immune‐mediated diseases, both with and without skin manifestations [10, 11]. Despite its clinical burden, no FDA‐approved treatments were available for AA prior to 2022 [2, 12]. In June 2022, baricitinib, a Janus kinase (JAK) inhibitor, was approved by the FDA for the treatment of severe AA in adults [13]. Subsequently, in June 2023, ritlecitinib, another JAK inhibitor, received FDA approval for the treatment of AA in both adolescents and adults [14, 15]. Most recently, in July 2024, deuruxolitinib was approved for the treatment of severe AA in adults [16].

Systemic steroids are sometimes used in AA treatment but are less favored due to their potential for serious side effects and a high relapse rate [3, 12]. Consequently, many physicians are hesitant to use systemic steroids for AA, except in special cases [4].

Azathioprine (AZA), synthesized in 1959 from 6‐mercaptopurine (6‐MP), possesses both immunosuppressive and anti‐inflammatory properties [17]. It is an antimetabolite drug widely used as an immunomodulatory, immunosuppressant, and steroid‐sparing agent, playing a significant role in treating a range of autoimmune and inflammatory dermatological diseases, such as vitiligo, psoriasis, atopic dermatitis, photodermatoses, systemic lupus erythematosus, dermatomyositis, immunobullous disorders, and vasculitides [18, 19, 20, 21]. However, it is only approved by the FDA for organ transplantation and severe rheumatoid arthritis. The exact mechanism of AZA action is unknown [17].

Our recent study demonstrated that AZA is a safe and effective treatment for AA over a 6‐month period [20]. In this article, we report the long‐term effectiveness and safety profile of AZA in 63 patients who received the drug for 10 years.

## 2 Patients and Methods

This study is a prospective study that followed patients over a 10‐year period. The study protocol adhered to the guidelines of the 1975 Declaration of Helsinki and was approved by the Medical Ethics Committee of Tehran University of Medical Sciences (approval number: 88‐04‐146‐18436). Written informed consent was obtained from all patients in their local language prior to enrollment.

Subjects with AA were recruited from the dermatology clinic of Imam Hospital, Tehran University of Medical Sciences, as well as the skin and stem cell research center.

Inclusion criteria for this study were:

- A history of AA for more than 6 months, ranging from patchy baldness to alopecia totalis and universalis.
- Nonscarring alopecia patches covering 20% or more of the scalp area.
- Patients who had not received any treatment for at least 1 month before the start of the study.

Exclusion criteria included:

- Sensitivity to AZA.
- Low levels of TPMT (thiopurine methyltransferase) activity.
- Concurrent therapy with other AA treatments, including topical therapies (steroids, tacrolimus, pimecrolimus), intralesional steroids, or systemic treatments (oral steroids, tofacitinib, cyclosporine, methotrexate, etc.).
- Use of other treatments affecting hair growth (e.g., minoxidil).
- Phototherapy treatments, including PUVA (Psoralen Ultraviolet A) and NBUVB (Narrow Band Ultraviolet B).
- Myelosuppressive disorders.
- Pregnancy and breastfeeding.
- Malignancy and other chronic diseases.

During the study, the patients did not receive any supplement that could affect hair growth. Routine laboratory tests were conducted before the start of the study, including complete blood count (CBC) with differentials, erythrocyte sedimentation rate (ESR), blood urea nitrogen (BUN), creatinine (Cr), liver function tests (LFT) including hepatic transaminases (ALT and AST), prothrombin time (PT), international normalized ratio (INR), partial thromboplastin time (PTT), thyroid function tests (TFT, including T3, T4, TSH), PPD or IGRA (Interferon Gamma Release Assay), and hepatitis tests (HBS Ag, HBS Ab, HBC Ab, HCV Ab). A chest X‐ray was also taken before the study began. TPMT activity was measured in all patients before and during treatment.

The daily dose of AZA was calculated as 2 mg/kg of body weight. Patients underwent laboratory tests every 2 months, including CBC, LFT, and PT. Patients who tested positive for PPD received 300 mg/day isoniazid and vitamin B6 in addition to AZA treatment for 6 months, with repeated assessments over the 10‐year period under the supervision of an infectious disease specialist.

### 2.1 Clinical Assessment

At the first visit, the patients' medical history was recorded, and an assessment of scalp involvement was performed. High‐resolution digital photographs were taken of each subject's treatment areas before treatment (baseline), at Months 1, 2, 3, 6, and then every 2 months for 10 years. Photos were taken from five different views of the scalp: the front, right and left temporal areas, vertex, and occipital regions.

Scalp assessment was conducted using the Severity of Alopecia Tool (SALT) score, with further classification of percent scalp hair loss into the following SALT subclasses: S0 = no hair loss, S1 = < 25% scalp hair loss, S2 = 26%–50% scalp hair loss, S3 = 51%–75% scalp hair loss, S4a = 76%–95% scalp hair loss, S4b = 96%–99% scalp hair loss, and S5 = 100% scalp hair loss.

Hair regrowth rate was assessed based on changes in the SALT score from baseline and further classified using the SALT scoring system: A0 = no change or further loss, A1 = 1%–24% regrowth, A2 = 25%–49% regrowth, A3 = 50%–74% regrowth, A4 = 75%–99% regrowth, A5 = 100% regrowth [2].

Changes in the SALT score were assessed during monthly visits. Adverse events were recorded throughout the study, and monthly laboratory assessments were conducted. Global photo ratings and SALT scores were determined at subsequent intervals.

### 2.2 Statistical Analysis

Statistical analysis was performed using IBM SPSS Statistics, version 25 (Chicago, IL, USA). A two‐tailed *p* value of 0.05 or less was considered statistically significant. Paired *t*‐tests were used to compare percentage hair loss and regrowth before treatment and after 10 years, as the data were normally distributed. Normality was tested using the Kolmogorov–Smirnov test. The SALT and regrowth scores were compared using the Wilcoxon signed‐rank test. Repeated measures analysis was used to assess percentage hair loss and regrowth over the 10‐year period. Missing data were handled using multiple imputation regression methods on the available data.

## 3 Results

The baseline characteristics of the participants are summarized in Table 1. The mean age of the patients was 24.62 ± 11.15 years, with a range of 8 to 59 years. The mean duration of the disease was 36.58 ± 46.28 months for male patients and 30.78 ± 25.77 months for female patients, with no statistically significant difference between the two groups (95% CI: −2.13 to 13.75). The mean age at the onset of the first episode was 15.53 ± 9.55 years for males and 14.26 ± 13.08 years for females, with a range of 2 to 54 years, again showing no statistically significant difference by gender (95% CI: −1.02 to 3.56).

**TABLE 1:** Baseline patient characteristics.

Variable	(*n* = 63)
Age (years), mean ± SD	24.62 ± 11.15
Sex, *n* (%)	
Male	36 (57.1)
Female	27 (42.9)
Age at onset of first episode of alopecia areata, mean ± SD	14.98 ± 11.28
Duration of alopecia (months), mean ± SD	34.1 ± 39.16
Nail involvement, *n* (%)	
Yes	28 (44.4)
No	35 (55.6)
Family history of Alopecia, *n* (%)	
Yes	22 (34.1)
No	41 (65.1)
History of thyroid disease, *n* (%)	
Yes	6 (9.5)
No	57 (90.5)
Scalp hair loss before treatment (%)	
< 25% hair loss	1 (1.6)
25%–49% hair loss	15 (23.8)
50%–74% hair loss	9 (14.3)
75%–95% hair loss	19 (30.2)
96%–99% hair loss	14 (22.2)
100% hair loss	5 (7.9)

**FIGURE 1:**
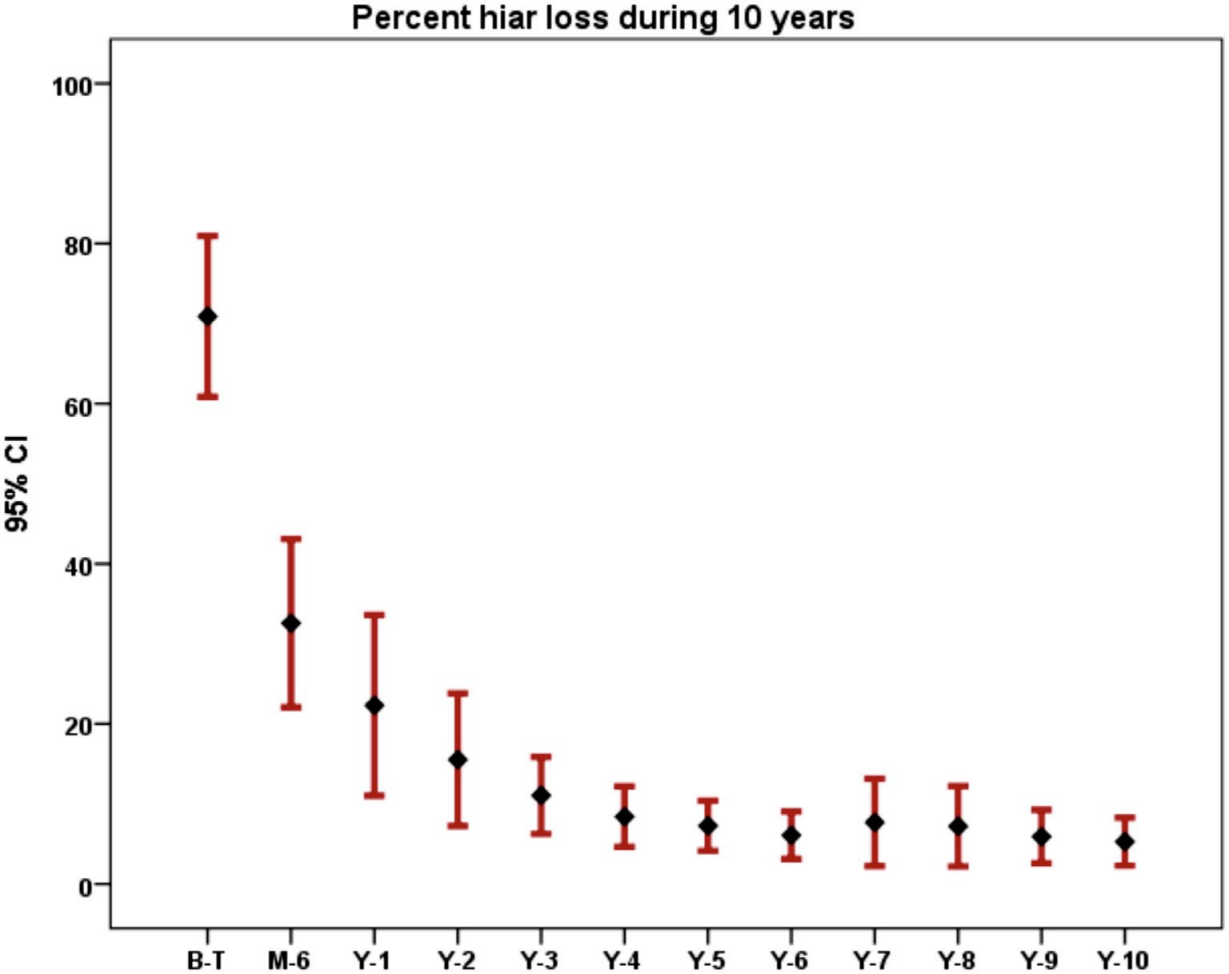
Error bar of percent hair loss for 10 years.

Table 2 presents the mean percentage of hair loss and the mean SALT scores over the 10‐year period. Data are provided both for the original dataset and after addressing missing data. Repeated measures analysis indicated that the differences in percentage hair loss over the 10‐year period were statistically significant (*p* = 0.0001). An error bar depicting the percentage of hair loss during the 10‐year follow‐up is shown in Figure 1.

**TABLE 2:** Percent hair loss and SALT scores before treatment and during 10‐year follow‐up of patients in original data and after analysis of missing data.

Original data	Percent hair loss before	6 M	1 Y	2 Y	3 Y	4 Y	5 Y	6 Y	7 Y	8 Y	9 Y	10 Y
Mean	74.21	44.12	31.11	25.51	20.56	15.24	15.8	9.91	10.95	9.25	8.43	5.29
SD	27.85	34.81	34.54	31.16	25.1	20.41	23.14	16.81	19.68	17.16	18.26	8.61
*N*	63	62	63	63	55	52	49	42	40	38	36	34
After imputation												
Mean	74.21	44.12	31.11	25.51	20.77	15.14	15.71	9.65	10.78	9.33	8.47	5.38
SD	27.85	34.55	34.54	31.16	23.52	18.59	20.5	13.82	15.85	13.52	14.03	6.59
*N*	63	63	63	63	63	63	63	63	63	63	63	63
Original data	SALT before											
Mean	3.71	2.27	1.78	1.6	1.36	1.1	1.12	0.93	0.93	0.87	0.92	0.68
SD	1.31	1.42	1.5	1.39	1.02	0.88	0.93	0.78	0.86	0.81	0.91	0.53
*N*	63	62	63	63	55	52	49	42	40	38	36	34
After imputation												
Mean	3.71	2.28	1.78	1.6	1.37	1.1	1.12	0.89	0.95	0.9	0.91	0.73
SD	1.31	1.4	1.5	1.39	0.98	0.83	0.94	0.76	0.85	0.75	0.87	0.61
*N*	63	63	63	63	63	63	63	63	63	63	63	63

Abbreviations: M, Month; *N*, Number of patients; SALT, Severity of Alopecia Tool; SD, Standard deviation; Y, Year.

The mean percentage of hair loss in male patients at baseline, Month 6, and Years 1 through 10 was 72.45 ± 26.88, 41.63 ± 31.28, 26.45 ± 32.51, 22.86 ± 29.72, 18.94 ± 25.72, 10.21 ± 11.47, 9.70 ± 11.09, 6.43 ± 5.81, 6.72 ± 5.85, 5.95 ± 6.03, 5.38 ± 5.39, and 4.71 ± 4.92, respectively. For female patients, the corresponding values were 76.54 ± 28.58, 47.44 ± 37.93, 37.31 ± 35.72, 29.04 ± 32.27, 22.76 ± 20.06, 21.44 ± 23.98, 23.87 ± 26.98, 14.80 ± 19.64, 16.33 ± 22.75, 13.20 ± 19.34, 11.83 ± 20.74, and 6.34 ± 8.67. No statistically significant difference was observed between male and female patients at baseline, Month 6 (95% CI: −9.75 to 1.54), Year 2 (95% CI: −12.83 to 1.2), and Year 3 (95% CI: −8.51 to 0.86). However, significant differences were found at Year 1 (95% CI: −17.80 to −3.93), Year 4 (95% CI: −14.96 to −7.5), and each subsequent year up to Year 10 (95% CI ranging from −18.24 to −0.18).

Table 3 details the mean regrowth percentage and mean SALT scores for hair regrowth during the 10‐year follow‐up, with data presented for both the original dataset and after missing data analysis. Repeated measures analysis showed statistically significant differences in mean regrowth rate over the 10‐year period (*p* = 0.0001).

**TABLE 3:** Regrowth percent and regrowth SALT scores during 10 years follow‐up in original data and after analysis of missing data.

Original data	Regrowth percent after 6 M	1 Y	2 Y	3 Y	4 Y	5 Y	6 Y	7 Y	8 Y	9 Y	10 Y
Mean	43.49	60.96	67.09	73.57	79.68	79.59	87.6	86.5	88.12	89.46	92.69
SD	36.36	36.26	34.57	28.12	24.05	25.55	16.96	20.04	17.89	18.08	9.08
*N*	62	63	63	55	52	49	42	40	38	36	34
After imputation											
Mean	43.53	60.96	67.09	73.59	79.68	79.92	87.57	86.49	88.08	89.64	92.92
SD	35.88	36.26	34.57	26.27	21.89	22.61	14.26	16.47	14.39	14.24	7.12
*N*	63	63	63	63	63	63	63	63	63	63	63
Original data	Regrowth SALT after 6 M										
Mean	2.21	2.97	3.16	3.44	3.75	3.82	4.1	4.03	4.11	3.97	4.32
SD	1.49	1.61	1.49	1.2	1.01	1.01	0.69	0.89	0.83	1.08	0.53
*N*	62	63	63	55	52	49	42	40	38	36	34
After imputation											
Mean	2.21	2.97	3.16	3.39	3.75	3.81	4.07	3.99	4.02	3.88	4.34
SD	1.49	1.61	1.49	1.14	0.96	0.98	0.86	0.82	0.82	0.99	0.55
*N*	63	63	63	63	63	63	63	63	63	63	63

Abbreviations: M, Month; *N*, Number of patients; SALT, Severity of Alopecia Tool; SD, Standard deviation; Y, Year.

In male patients, the mean regrowth rates at Month 6 and Years 1 through 10 were 46.10 ± 32.67, 66.56 ± 34.96, 70.04 ± 33.98, 75.47 ± 28.86, 85.30 ± 14.39, 87.06 ± 12.98, 91.19 ± 7.10, 90.29 ± 8.47, 91.28 ± 8.61, 92.50 ± 6.15, and 94.03 ± 5.56, respectively. In female patients, these values were 40.07 ± 39.62, 53.51 ± 36.17, 63.16 ± 34.53, 71.05 ± 22.44, 70.32 ± 28.99, 82.64 ± 19.29, 81.31 ± 22.35, 83.72 ± 18.92, 85.64 ± 20.15, and 91.37 ± 8.64. No statistically significant differences were found between male and female patients at Month 6 (95% CI: −1.30 to 13.36), Year 2 (95% CI: −0.11 to 13.87), and Year 3 (95% CI: −1.03 to 9.86). However, significant differences were observed at Year 1 (95% CI: 5.80 to 20.30) and from Year 4 to Year 10 (95% CI ranging from 1.16 to 21.18).

**FIGURE 2:**
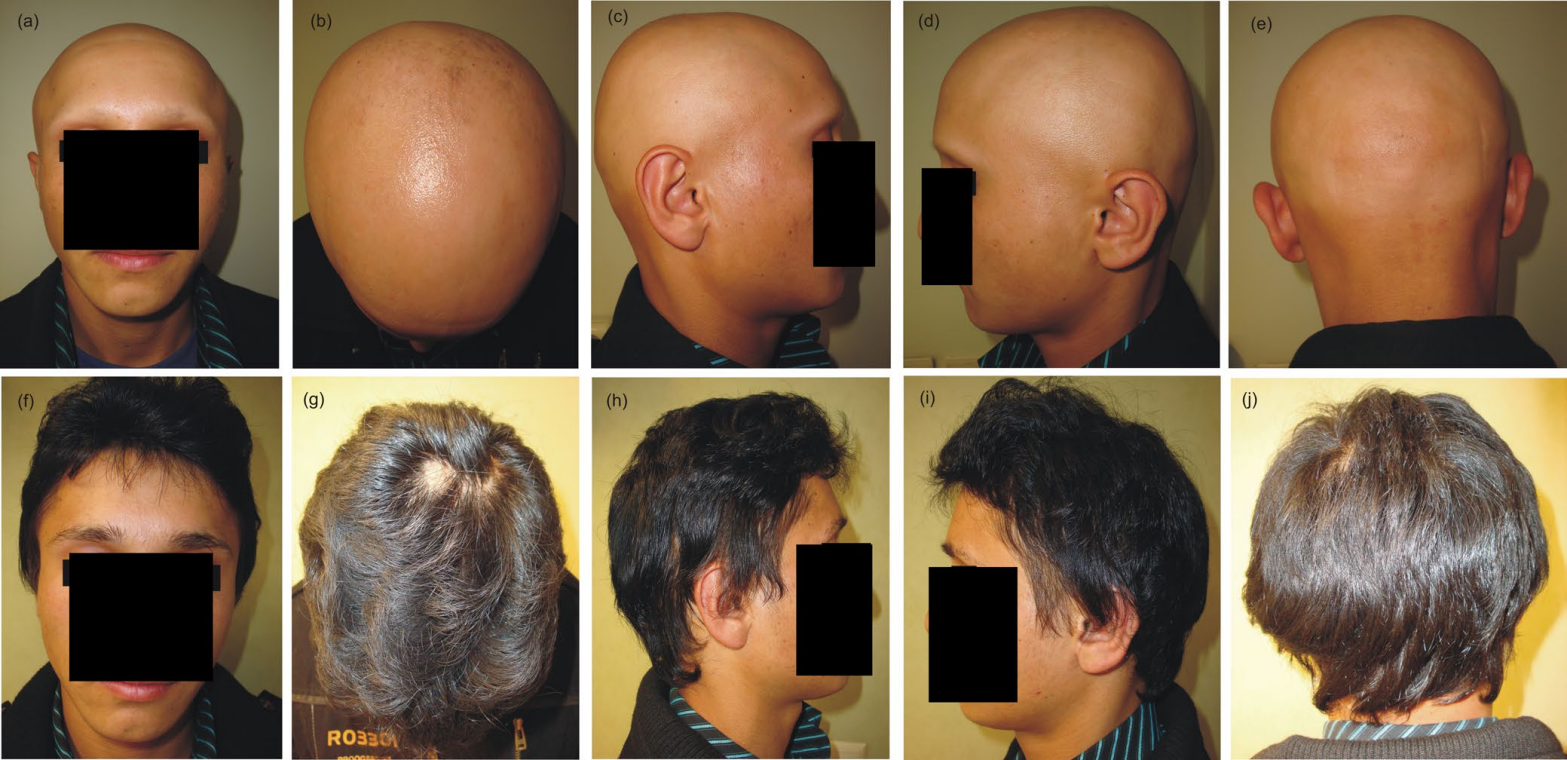
A 27‐year‐old man before treatment (a– e) and after receiving azathioprine for 2 years (f–j).

A good clinical response in one patient after 2 years of follow‐up is shown in Figure 2. Twenty‐two patients had no hair loss in their eyelashes, and 16 patients had no hair loss in their eyebrows. The mean percentage of hair in the eyelash and eyebrow regions before treatment was 50.87 ± 44.31 and 46.54 ± 42.40, respectively. After 10 years, these values improved to 67.68 ± 41.38 in the eyelash region and 76.52 ± 32.92 in the eyebrow region.

Three patients tested positive for PPD and received isoniazid (300 mg daily) and vitamin B6 for 6 months. One patient experienced complete scalp regrowth after the first year of treatment, though all hair turned gray and white. After another year, the hair color returned to black. However, this patient experienced total hair loss again in the 8th year and discontinued treatment after 9 years, opting for tofacitinib.

A male patient died in a car accident during the 6th year of follow‐up after achieving near‐complete hair regrowth. Most patients who did not respond adequately to AZA by the end of the 3rd or 4th year chose to discontinue treatment. One female patient became pregnant after 5 years and was excluded from the study but resumed treatment postpartum. A patient with Down syndrome did not return after 3 years, and follow‐up attempts were unsuccessful. One male patient who completed 10 years of AZA treatment did not experience hair loss 5 years after stopping the drug.

Adverse events were closely monitored throughout the study. Most adverse events were mild and transient. Two female patients developed mild anemia, which resolved after reducing the drug dosage. Mild leukopenia was reported in one male patient after 3 years of AZA therapy, which returned to normal with a slight dose reduction. Hair regrowth in the beard and body hair of male patients was not systematically evaluated but was observed in many patients with facial and body involvement.

## 4 Discussion

AA can affect individuals of any age, with the clinical course varying from self‐limiting episodes to chronic, relapsing, and recalcitrant disease [21]. Because it has a significant likelihood of natural remission, particularly in the early stages of the disease, the interpretation of a successful treatment response is complicated, especially when the number of participants in each arm of the study is so small [3]. However, spontaneous hair regrowth is rare in patients with long‐standing AA [20].

AZA, developed over five decades ago, has been used successfully in the treatment of various inflammatory and autoimmune diseases [17, 22].

Although there are few studies reporting the effects of AZA in treating AA, existing data suggest promising results [20, 21, 23]. For instance, one study on 14 patients with recalcitrant AA showed that AZA was effective in 43% of cases [23]. In our previous study, the mean regrowth rate was 52.3% [20], whereas in the present study, the mean regrowth rate was significantly higher at 92.69%. Although the differing evaluation methods in these studies prevent direct comparison, the higher regrowth rate observed in our study suggests that AZA may be more effective over the long term.

Sood et al. reported that AZA was well tolerated, with only 16.22% of patients experiencing side effects that required discontinuation [24]. This finding aligns with our study, where AZA was similarly well tolerated.

The mechanism of AZA‐induced liver toxicity remains unclear but is believed to involve elevated levels of 6‐MMP (6‐methylmercaptopurine) due to high TPMT (thiopurine methyltransferase) activity. Prolonged exposure to high 6‐MMP levels may contribute to hepatotoxicity [25], whereas low TPMT activity can lead to hematotoxic side effects due to elevated levels of 6‐thioguanine. Bone marrow toxicity occurs in about 2%–5% of patients treated with AZA [26].

Elevated liver enzymes during AZA treatment have been reported in 2%–3% of patients. Most of these improve with dose reduction or drug discontinuation [26]. In our study, patients with normal TPMT levels were included, resulting in minimal hematological complications. Mild and transient liver enzyme elevation was observed in some patients, which improved with dose reduction.

In contrast to our previous study, where TPMT levels were not measured, resulting in some cases of decreased white blood cell counts, the current study's pre‐screening for TPMT levels likely contributed to the absence of significant hematological complications. In a study by Gupta et al., pulse therapy with AZA 300 mg per week showed no significant difference in efficacy compared to betamethasone 5 mg on two consecutive days per week, though AZA had a lower rate of adverse events, suggesting a better safety profile [27].

A study showed that methotrexate (MTX, 15–25 mg per week) alone or in combination with 10–20 mg per day of prednisone resulted in complete regrowth in 57% and 63% of patients, respectively; however, the relapse rate was 80%. Hair regrowth was observed after an average delay of 3 months [12]. This result was the same in the delay action of AZA in our studies, which was 3–6 months [20]. Although combining MTX with AZA has shown encouraging results in severe and recalcitrant AA cases [28], the high rate of adverse events limits its use [29].

A study comparing the effect of AZA and mesalazine in the treatment of severe AA showed the same effects [30].

Recent studies have highlighted the effectiveness of biologic drugs and JAK inhibitors, such as tofacitinib and baricitinib, in treating AA [31, 32, 33, 34]. However, the high cost and limited availability of these drugs in many countries make them inaccessible for long‐term treatment.

Our findings suggest that the regrowth rate in male patients was higher than in female patients over 10 years. Although no difference was observed between genders in the first 6 months, or in Years 2 and 3, a significant difference emerged over the longer follow‐up period. Additionally, our data indicate that eyebrow hairs respond better to AZA treatment than eyelash hairs, though further studies are needed to confirm this observation.

The significant and sustained hair regrowth observed over 10 years is a remarkable result, even after accounting for missing data. However, the true regrowth rate may be lower, as some patients may have left the study due to inadequate response or relapse, which is one of the study's limitations. Other limitations include the nonblinded design and the lack of a control group.

In conclusion, our 10‐year follow‐up suggests that AZA is a safe and well‐tolerated treatment for AA. We hope that future double‐blind, controlled clinical trials will further clarify AZA's effectiveness compared to other treatments in patients with severe and recalcitrant AA.

## Author Contributions

Conceptualization: Parvin Mansouri, Susan Farshi. Data curation: Susan Farshi, Parvin Mansouri. Formal analysis: Susan Farshi. Investigation: Parvin Mansouri, Susan Farshi. Methodology: Susan Farshi. Project administration: Susan Farshi. Resources: Parvin Mansouri, Susan Farshi. Software: Susan Farshi. Supervision: Parvin Mansouri. Validation: Susan Farshi, Parvin Mansouri. Visualization: Susan Farshi. Writing – original draft preparation: Susan Farshi, Parvin Mansouri. Writing – review and editing: Susan Farshi, Parvin Mansouri.

## Ethics Statement

The study protocol conformed to the guidelines of the 1975 Declaration of Helsinki and was approved by the Medical Ethics Committee of Tehran University of Medical Sciences with the approval number of 88‐04‐146‐18436. None of the authors is employed by the Iranian government sector.

## Conflicts of Interest

The authors declare no conflicts of interest.

## Data Availability

The data that support the findings of this study are available from the corresponding author upon reasonable request.
